# RETRACTION: Improvement of chemotherapeutic drug efficacy by endoplasmic reticulum stress

**DOI:** 10.1530/ERC-15-0019r

**Published:** 2024-10-18

**Authors:** Chrysovalantou Mihailidou, Ioulia Chatzistamou, Athanasios G Papavassiliou, Hippokratis Kiaris

**Affiliations:** 1 Department of Biological Chemistry, University of Athens Medical School, Athens Greece; 2 Department of Basic Sciences, Dental School, University of Athens, Athens Greece; 3 Department of Pathology, Microbiology and Immunology, University of South Carolina School of Medicine, Columbia, South Carolina, USA; 4 Department of Drug Discovery and Biomedical Sciences, University of South Carolina, Columbia, South Carolina, USA

This article has been retracted by the journal *Endocrine-Related Cancer*.

The article, ‘Improvement of chemotherapeutic drug efficacy by endoplasmic reticulum stress’ published on 23 March 2015, volume 22 pages 229–238, has been retracted due to irregularities in the data provided in Figure 1 that affect confidence in the conclusions of the paper. *Endocrine-Related Cancer* was made aware of, and agreed with, the concerns raised regarding the data.

Three of the authors (Hippokratis Kiaris, Ioulia Chatzistamou and Athanasios Papavassiliou) requested and agreed with the retraction decision. The first author, Chrysovalantou Mihailidou, who performed the corresponding experiments, remained out of reach to address these issues. In view of the doubts expressed the article has now been retracted. No further action will be taken against the authors of the article.

This retraction was published on 18 October 2024.

